# Risk of cardiovascular events among patients with HIV treated with atazanavir-containing regimens: a retrospective cohort study

**DOI:** 10.1186/s12879-016-1827-1

**Published:** 2016-09-19

**Authors:** Lisa Rosenblatt, Amanda M. Farr, Ella T. Nkhoma, James K. Nelson, Corey Ritchings, Stephen S. Johnston

**Affiliations:** 1 Bristol-Myers Squibb, 777 Scudders Mill Road, Plainsboro, NJ 08536 USA; 2 Truven Health Analytics, 150 Cambridgepark Drive, Cambridge, MA 02140 USA; 3 Bristol-Myers Squibb, 5 Research Parkway, Wallingford, CT 06492 USA; 4 Truven Health Analytics, 100 Phoenix Drive, Ann Arbor, MI 48108 USA; 5 Bristol-Myers Squibb, PO Box 4500, Princeton, NJ 08540 USA; 6 Truven Health Analytics, 7700 Old Georgetown Road, Bethesda, MD 20814 USA

**Keywords:** Human immunodeficiency virus, Anti-retroviral agents, Protease inhibitors, Atazanavir, Major adverse cardiovascular events

## Abstract

**Background:**

A previous cohort study indicated that atazanavir (ATV), a protease inhibitor used for HIV treatment, is not associated with an increased risk of cardiovascular (CV) events. The objective of this study was to compare the risk of CV events among antiretroviral-naïve patients initiating ATV-containing versus ATV-free ARV regimens.

**Methods:**

Patients with HIV who newly initiated antiretroviral therapy were selected from MarketScan Commercial and Multi-State Medicaid databases. The first claim for an antiretroviral medication between 1/1/2007 and 12/31/2013 was known as the index date. Patients were categorized as initiating an ATV-containing or an ATV-free regimen. Patients who did not have 6 months of continuous enrollment prior to the index date or who had evidence of a CV event during this time period were excluded. Myocardial infarction, stroke, percutaneous coronary intervention, and coronary artery bypass graft were identified through diagnosis and procedure codes. Patients were followed from index date until a CV event, continuous gap of >30 days without initiated ARV, a claim for ATV in the ATV-free cohort, disenrollment, or study end, whichever occurred first. Unadjusted incidence rates (IR) were calculated and propensity-score-weighted Cox proportional hazards models were fit to compare hazards of CV events between the two cohorts.

**Results:**

A total of 22,211 patients (2437 ATV-containing and 19,774 ATV-free) were identified in the Commercial Database and 7136 patients were identified (1505 ATV-containing and 5631 ATV-free) in the Medicaid Database. CV events were uncommon (Commercial IR per 1000 person-years for a CV event: ATV-containing = 3.01, ATV-free = 3.26; Medicaid IR: ATV-containing = 10.9, ATV-free = 9.9). In propensity-score-weighted models combining the two populations, there was no significant difference in the hazards of a CV event for patients initiating an ATV-containing regimen compared with those initiating an ATV-free regimen (hazard ratio = 1.16, 95 % confidence interval 0.67–1.99).

**Conclusions:**

In this real-world analysis, there was no significant increase in the risk of CV events associated with exposure to ATV-containing regimens.

**Electronic supplementary material:**

The online version of this article (doi:10.1186/s12879-016-1827-1) contains supplementary material, which is available to authorized users.

## Background

Cardiovascular (CV) disease is of clinical importance among individuals with HIV. Several analyses have reported that risk of CV disease is higher among people with HIV compared to uninfected individuals, even after adjusting for traditional risk factors [1–4]. Use of certain antiretroviral (ARV) medications used to treat HIV infection have been implicated in this increased risk [1, 5]. Analyses of older data have found that use of protease inhibitors (PIs), one class of antiretroviral medications, is associated with increased risk for CV events [1, 5]. One large prospective study, the Data Collection on Adverse Events of Anti-HIV Drugs (D:A:D), reported that use of PIs was associated with myocardial infarction (MI), due in part to dyslipidemia, based on data collected through 2005 [6]. A systematic review and meta-analysis using analyses published through 2011 found that cumulative exposure to PIs, particularly indinavir and lopinavir, was also associated with increased risk of MI [5].

However, there is not as much data available with more recently approved PIs, and the available studies have not found the same association with CV disease. An analysis evaluating atazanavir (ATV) use and risk for MI or stroke in the D:A:D cohort reported no significant association with either outcome [7]. Additionally, a recent randomized clinical trial reported that treatment-naïve patients who initiated ritonavir-boosted ATV (ATV/r) had significantly slower progression of atherosclerosis over a 3-year period compared to patients treated with ritonavir-boosted darunavir (DRV/r) [8]. These patients also had slower progression compared with patients treated with raltegravir, though the difference was only statistically significant at the carotid bifurcation and in the on-treatment analyses [8]. In combination, these analyses suggest that not all medications within the PI class have the same CV risk profile.

In order to confirm these findings, the primary objective of this claims-based analysis was to compare incidence rates and hazards of CV events between antiretroviral-naïve HIV+ patients initiating ATV-containing versus ATV-free ARV regimens. The secondary objectives were to compare these outcomes between HIV+ patients initiating ATV-containing regimens versus (a) PI-free regimens, (b) other PI-containing regimens, and (c) darunavir (DRV)-containing regimens.

## Methods

### Data sources

The Truven Health MarketScan® Commercial Claims and Encounters (Commercial) and Multi-State Medicaid (Medicaid) insurance claims databases were used to conduct this analysis. The databases contain inpatient and outpatient medical claims, outpatient prescription drug claims, and enrollment information of enrollees from a variety of fee-for-service and managed care health plans from a convenience sample of over 300 large self-insured employers and over 25 health plans, and similar claims from Medicaid insurance from 15 geographically dispersed states in the US. Patients are included in the database if their health insurer is one of the data contributors to MarketScan. They remain in the database until they become unenrolled. For example, if a patient has insurance through his/her employer and the employer is a contributor to MarketScan, that patient would be included in the MarketScan enrollment files until he/she switched employers. If the patient was with that employer for 3 years, all healthcare claims generated by the patient in those 3 years (i.e., office visits, prescriptions, etc.) would appear in the MarketScan database.

There are approximately 138 million enrollees in the Commercial database and approximately 29 million enrollees in the Medicaid database. All records contained within the databases are statistically de-identified and fully compliant with the conditions designated by the Health Insurance Portability and Accountability Act (HIPAA) Privacy Regulations. Institutional Review Board approval was not sought, as the data did not contain any individually identifiable patient information.

### Patient selection

Patients with at least one prescription claim with a *National Drug Code* for ATV, another PI, a non-nucleoside reverse transcriptase inhibitor (NNRTI), an integrase inhibitor, a fusion inhibitor, or a CCR5 antagonist between January 1, 2007 and December 31, 2013 were selected from the Commercial and Medicaid databases. The date of first claim was known as the index date and the medication filled on that date was defined as the index drug. The following inclusion criteria were then applied to the initial group of patients: age 18–64 on the index date, continuous enrollment for at least six months prior to the index date, no evidence of a CV event of interest (MI, stroke, percutaneous coronary intervention [PCI], or coronary artery bypass graft [CABG]) in the six months prior to the index date, at least one medical claim with a diagnosis of HIV infection (*International Classification of Diseases, Ninth Edition, Clinical Modification* [ICD-9-CM] 042, V08, 795.71, 079.53) prior to index date using all available data starting in 2004, no claims for ARV medications prior to the index date using all available prior data starting in 2004 to attempt to ensure patients were treatment-naïve, and no dual eligibility for Medicare. The final patient samples were categorized into two cohorts based on ARV claims on the index date for the primary analysis: ATV-containing regimen cohort or ATV-free regimen cohort. To address the secondary objectives, patients initiating ATV-free regimens were further categorized as initiating PI-free regimens, other PI-containing regimens, and DRV-containing regimens. Full patient attrition is shown in Table 1.

**Table 1:** Patient attrition for antiretroviral-naïve HIV+ patients initiating atazanavir-containing vs. atazanavir-free regimens

Criterion	Commercial Database	Medicaid Database
Patients with ≥1 prescription for an ARV medication^a^ between 1/1/2007 and 12/31/2013 (first claim = index date)	98,672	31,806
AND age 18–64 at index date	97,911	29,782
AND continuous enrollment for ≥6 months prior to index date	36,942	13,197
AND no eligibility for Medicare	36,942	13,181
AND no claims for ARV medications prior to index date^b^	26,958	7,731
AND ≥1 claim with an HIV diagnosis prior to index date^b^	22,285	7,185
AND no CV event^c^ in the 6 months prior to index date	22,211	7,136
Final Study Population	22,211	7,136
Initiated ATV-containing regimen	2,437	1,505
Initiated ATV-free regimen	19,774	5,631
Initiated PI-free regimen	16,131	3,931
Initiated other PI-containing regimen	3,643	1,700
Initiated DRV-containing regimen	1,551	527

*ARV* antiretroviral, *ATV* atazanavir, *DRV* darunavir, *CV* cardiovascular, *PI* protease inhibitor

^a^ARV medications included non-nucleoside reverse transcriptase inhibitors, protease inhibitors (excluding ritonavir), integrase inhibitors, fusion inhibitors and CCR5 antagonists. ^b^Using all data prior to index date starting with 2004. ^c^Myocardial infarction, stroke, percutaneous coronary intervention, or coronary artery bypass graft

### Study time period

The study period consisted of a baseline period, index date, and a variable-length follow-up period. The baseline period, during which patient characteristics were measured, was the six months prior to the index date. As described above, the index date was the date of antiretroviral therapy (ART) initiation. Patients were followed from the index date to the occurrence of a CV event, a gap of >30 consecutive days without index drug, a claim for ATV for patients in the ATV-free cohort, disenrollment, or end of the available data. Follow-up was calculated separately for each individual CV event. Patients were not required to be on ARV medications for any pre-specified length of time during the follow-up period. In intent-to-treat (ITT) sensitivity analyses, patients were followed from index date until a CV event, end of continuous enrollment, or end of the study data, regardless of changes to ARV medications.

### CV outcomes

Medical claims during the follow-up period were evaluated for diagnosis or procedure codes indicative of the following CV events: MI, stroke, PCI, or CABG. Additionally, a composite CV event was captured which included any of the aforementioned events. The presence of an MI was determined by an inpatient medical claim with a diagnosis code for MI (ICD-9-CM 410.xx) recorded in the primary diagnosis position [9]. Similarly, stroke was defined as an inpatient medical claim with a diagnosis code for stroke (ICD-9-CM 430.xx, 431.xx, 434.x1, 436.xx) recorded in the primary diagnosis position [10]. Both definitions were based on previously published algorithms [9, 10]. PCI and CABG events were based on an inpatient or outpatient medical claim with an ICD-9-CM procedure, Current Procedure Terminology, or Healthcare Common Procedure Coding System codes for these procedures recorded in any position (Additional file 1: Table S1). At the patient level, the presence of each CV event and time from index date to first evidence of each CV event were captured. The number of each type of CV event experienced by a patient (i.e., the number of MIs that a single individual had during follow-up) was not captured.

### Covariates

Several demographic and clinical variables were captured. The full list of covariates is presented in Tables 2 and 3. Demographic characteristics were measured on the index date and included age, sex, region (available only in commercial data), and race (available only in Medicaid data). Clinical characteristics were captured during the baseline period. CV risk factors, such as CHADS_2_ score (a classification scheme used to predict stroke risk) [11, 12], hypertension, dyslipidemia, and circulatory disease were measured based on diagnoses on medical claims and prescription claims. Additionally, characteristics of the initiated ARV regimen were captured. Any ARV medication filled on or within 13 days of the index date was considered part of the initiated regimen. A sensitivity analysis was conducted on patients determined to be at high risk for a CV event. Patients were considered high risk if they met at least one of the following criteria: age 55–64, evidence of diabetes, evidence of hypertension, evidence of dyslipidemia, evidence of tobacco use disorder, or evidence of circulatory disease.

**Table 2:** Demographic characteristics of antiretroviral-naïve HIV+ patients initiating atazanavir-containing vs. atazanavir-free regimens

	Commercial Database					Medicaid Database				
	ATV-Containing Regimen *N* = 2,437		ATV-Free Regimen *N* = 19,774		*p*-value	ATV-Containing Regimen *N* = 1,505		ATV-Free Regimen *N* = 5,631		*p*-value
	N	%	N	%		N	%	N	%	
Age in Years (Mean, SD)	41.0	10.2	40.4	10.5	0.0055	41.2	10.9	41.3	11.2	0.6919
Male	1,863	76.4 %	16,566	83.8 %	<.0001	746	49.6 %	2,883	51.2 %	0.2282
Region^a^										
Northeast	439	18.0 %	3,146	15.9 %	0.0002					
North Central	290	11.9 %	2,896	14.6 %						
South	1,229	50.4 %	10,174	51.5 %						
West	440	18.1 %	3,300	16.7 %						
Unknown	39	1.6 %	258	1.3 %						
Race^b^										
White						206	13.7 %	964	17.1 %	0.0153
Black						1,088	72.3 %	3,894	69.2 %	
Hispanic						16	1.1 %	76	1.3 %	
Other						13	0.9 %	62	1.1 %	
Unknown/Missing						182	12.1 %	635	11.3 %	
Capitation^c^	548	22.5 %	3,754	19.0 %	<.0001	609	40.5 %	2,256	40.1 %	0.7779

*ATV* atazanavir, *SD* standard deviation

^a^Commercial population only; ^b^Medicaid population only; ^c^Presence of claim with capitated payment arrangement

**Table 3:** Baseline clinical characteristics of antiretroviral-naïve HIV+ patients initiating atazanavir-containing vs. atazanavir-free regimens

	Commercial Database					Medicaid Database				
	ATV-Containing Regimen *N* = 2,437		ATV-Free Regimen *N* = 19,774		*p*-value	ATV-Containing Regimen *N* = 1,505		ATV-Free Regimen *N* = 5,631		*p*-value
	N	%	N	%		N	%	N	%	
CHADS_2_ Score^a^										
0	2,040	83.7 %	16,382	82.8 %	0.3593	1,019	67.7 %	3,745	66.5 %	0.6718
1	321	13.2 %	2,734	13.8 %		330	21.9 %	1,294	23.0 %	
2	67	2.7 %	535	2.7 %		113	7.5 %	448	8.0 %	
3–6	9	0.4 %	123	0.6 %		43	2.9 %	144	2.6 %	
Diabetes Mellitus^b^	115	4.7 %	961	4.9 %	0.7597	137	9.1 %	563	10.0 %	0.2996
Hypertension^b^	370	15.2 %	3,256	16.5 %	0.1058	450	29.9 %	1,733	30.8 %	0.5125
Dyslipidemia^b^	306	12.6 %	2,473	12.5 %	0.9438	158	10.5 %	576	10.2 %	0.7600
Renal Disease	107	4.4 %	640	3.2 %	0.0029	146	9.7 %	524	9.3 %	0.6404
Tobacco Use Disorder	110	4.5 %	986	5.0 %	0.3095	347	23.1 %	1,257	22.3 %	0.5448
COPD	37	1.5 %	279	1.4 %	0.6730	88	5.8 %	392	7.0 %	0.1253
Anemia	186	7.6 %	1,710	8.6 %	0.0905	283	18.8 %	1,088	19.3 %	0.6507
Hepatitis C	67	2.7 %	419	2.1 %	0.0448	173	11.5 %	595	10.6 %	0.3018
Alcohol Abuse Disorder	14	0.6 %	147	0.7 %	0.3536	90	6.0 %	246	4.4 %	0.0088
Drug Abuse Disorder	137	5.6 %	1,250	6.3 %	0.1780	488	32.4 %	1,666	29.6 %	0.0331
Autoimmune/Inflammatory Disorders	86	3.5 %	731	3.7 %	0.6779	80	5.3 %	284	5.0 %	0.6700
Circulatory Disease	504	20.7 %	4,470	22.6 %	0.0316	570	37.9 %	2,178	38.7 %	0.5686
Oral Contraceptives	25	1.0 %	146	0.7 %	0.1255	13	0.9 %	42	0.7 %	0.6422

*COPD* chronic obstructive pulmonary disorder, *ATV* atazanavir, *SD* standard deviation

^a^CHADS_2_ is based on the presence of diagnoses of congestive heart failure, hypertension, diabetes, and stroke or transient ischemic attack and age ≥ 75 [11, 12]; ^b^Both diagnoses and medication use were evaluated

### Statistical analysis

The commercial and Medicaid patient populations were analyzed separately in the descriptive analysis as the rates of CV events in each population was of interest. Patient characteristics were compared between the ATV-containing and ATV-free cohorts using t-tests for continuous variables and chi-squared tests for categorical variables. Unadjusted incidence rates (IR) and 95 % confidence intervals (CI) for each individual CV event and the composite CV event were calculated as the number of patients with the event divided by sum of person-time during follow-up. Following the descriptive analysis, the two patient populations were combined to increase statistical power. Propensity score weights were calculated using the covariates in Tables 2 and 3 to adjust for baseline differences between the ATV-containing and ATV-free cohorts. Two propensity scores were generated. The first was a local propensity score which included all variables available in each database and the second was a universal propensity score which included all variables that were available in both databases [13]. For example, the local score for Medicaid included race while the universal score did not, because race is not available in the commercial data. To compare hazards of CV events, propensity-score-weighted Cox proportional hazards models were fit. Patients who did not experience a CV event were considered censored at the end of follow-up. The same methods were used for the secondary comparisons and the sensitivity analyses.

## Results

### Patient characteristics

There were 98,672 patients in the commercial database and 31,806 in the Medicaid database who had at least one claim for an ARV medication (Table 1). After applying the inclusion and exclusion criteria, the final sample sizes were 22,211 and 7163 in the commercial and Medicaid databases, respectively. Many potential patients were excluded due to the pre-period continuous enrollment criteria. However, this criterion was necessary for appropriate assessment of baseline characteristics. In the commercial sample, approximately 11 % of patients initiated an ATV-containing regimen. There were significantly fewer males in the ATV-containing regimen cohort than the ATV-free cohort (Table 2). The proportions of patients with renal disease or hepatitis C were significantly larger in the ATV-containing cohort (Table 3). CHADS_2_ scores were similar between the two cohorts. In the Medicaid sample, approximately 21 % of patients initiated an ATV-containing regimen. The proportion of patients with white race was significantly lower in the ATV-containing regimen cohort (Table 2). Compared with the patients initiating ATV-free regimens, a significantly larger proportion of patients initiating ATV-containing regimens were previously diagnosed with alcohol or drug abuse (Table 3). Again, CHADS_2_ scores were similar between the two cohorts. In both databases, more than half of patients initiated a PI-free regimen. Of those patients, 74 % in both databases initiated efavirenz. Results comparing the demographic and baseline clinical characteristics between patients initiating ATV-containing regimens and those initiating PI-free regimens, other PI-containing regimens, and DRV-containing regimens are presented in Additional file 1: Tables S2 and S3.

For the primary analysis (as-treated), the mean follow-up for ATV-containing cohorts was 12 months (median = 5 months) for the commercial sample and 9 months (median = 4 months) for the Medicaid sample compared to mean follow-up of 13 months (median = 7 months) and 9 months (median = 4 months), respectively, for the ATV-free cohorts. For the ITT sensitivity analysis, the mean follow-up for ATV-containing cohorts was 24 months (median = 18 months) for the commercial sample and 23 months (median = 17 months) for the Medicaid sample compared to mean follow-up of 21 months (median = 16 months) and 22 months (median = 15 months), respectively, for the ATV-free cohorts.

### CV events

**Fig. 1:**
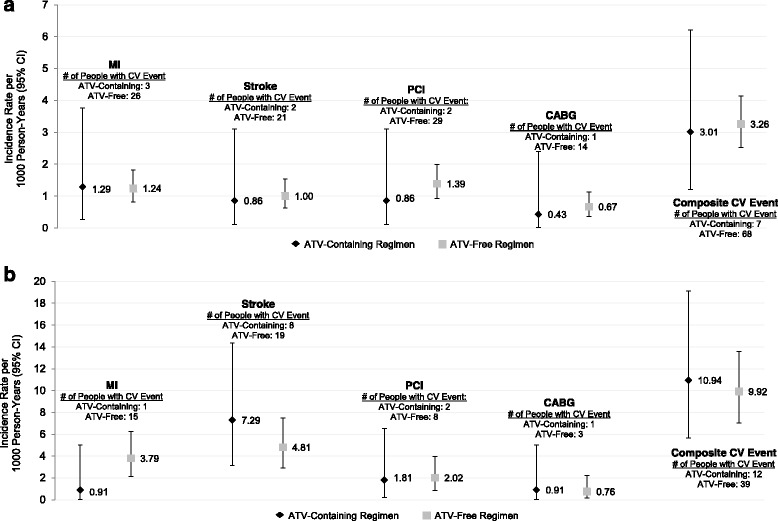
Incidence Rate per 1000 Person-Years for both **a** and **b**. Unadjusted incidence rates for cardiovascular events among commercially-insured (**a**) and Medicaid-insured (**b**) HIV+ patients. ATV, atazanavir; CABG, coronary artery bypass graft; CI, confidence interval; CV, cardiovascular; PCI, percutaneous coronary intervention. Note. A single individual may have had more than one type of CV event and therefore, the sum of the numbers of people with each individual event may be greater than the number of people with the composite CV event

CV events were rare in both databases. In the commercial sample, incidence rates for each individual CV event were less than 2 per 1000 person-years in both cohorts (Fig. 1a and Additional file 1: Table S4A). For the composite CV outcome, the incidence rates were similar for the ATV-containing cohort (IR per 1000 person-years = 3.01, 95 % CI 1.21, 6.21) and the ATV-free cohort (IR per 1000 person-years = 3.26, 95 % CI 2.53, 4.13). Patients initiating other PI-containing regimens tended to have higher incidence of CV events (IR for composite CV outcome per 1000 person-years = 6.63, 95 % CI 3.99, 10.36) (Additional file 1: Table S4A). Though still rare, CV events were more common among Medicaid patients than Commercial patients. IRs for the composite CV event outcome in the Medicaid database were 10.94 per 1000 person-years (95 % CI 5.66, 19.12) for patients initiating ATV-containing regimens and 9.92 per 1000 person-years (95 % CI 7.06, 13.56) for patients initiating ATV-free regimens (Fig. 1b and Additional file 1: Table S4B). IRs ranged from 9.85 to 10.87 among the sub-cohorts of the ATV-free regimens (Additional file 1: Table S4B).

**Fig. 2:**
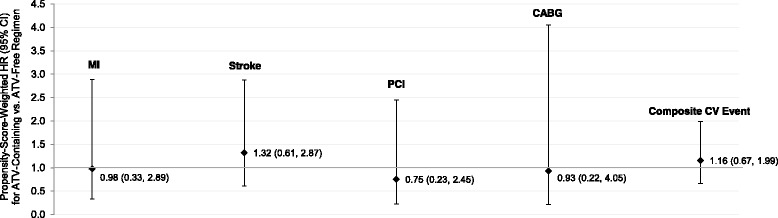
Propensity-score-weighted hazard ratios for CV events during as-treated follow-up period: Primary comparison. ATV, atazanavir; CABG, coronary artery bypass graft; CI, confidence interval; CV, cardiovascular; HR, hazard ratio; MI, myocardial infarction; PCI, percutaneous coronary intervention

**Fig. 3:**
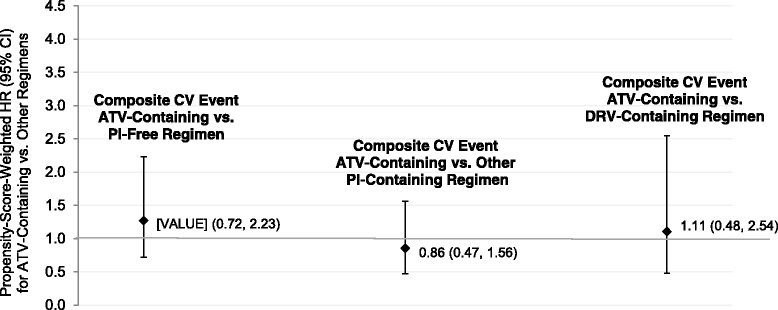
Propensity-score-weighted hazard ratios for composite CV event during as-treated follow-up period: Secondary comparisons. CI, confidence interval; CV, cardiovascular; DRV, darunavir; HR, hazard ratio; PI, protease inhibitor

After combining the commercial and Medicaid patients, hazard ratios weighted by the universal propensity score for CV events comparing ATV-containing regimens and ATV-free regimens were all not statistically significant (Fig. 2 and Additional file 1: Table S5). The hazard ratio for the composite CV outcome was 1.16 (95 % CI 0.67, 1.99). When limiting the sample to patients at high risk for CV events, there was no significant difference in the hazards of the composite CV outcome between ATV-containing and ATV-free regimens (hazard ratio = 1.16, 95 % CI 0.64-2.10). The hazard ratios comparing ATV-containing regimens with PI-free, other PI-containing, and DRV-containing regimens in terms of the composite CV outcome were also non-significant (Fig. 3 and Additional file 1: Table S5). The findings were similar with the individual CV events (Additional file 1: Table S5). Results weighted by the local propensity score were similar and have not been presented here. During the ITT follow-up period, unadjusted IRs were higher than they were for the as-treated follow-up periods but CV events were still rare (Additional file 1: Tables S4A and S4B). All propensity-score-weighted hazard ratios for comparisons were nonsignificant (Additional file 1: Table S5).

## Discussion

In this real-world analysis of HIV+ patients with commercial or Medicaid insurance, CV events were rare, though more common among Medicaid-insured patients. There was no significant increase in the risk of CV events associated with exposure to ATV-containing ARV regimens compared with ATV-free regimens. The findings were the same when using an as-treated or ITT follow-up. Additionally, there were no significant differences in risk of CV events between patients initiating ATV-containing regimens compared to PI-free regimens, other PI-containing regimens, or DRV-containing regimens. These results lend additional evidence to the body of literature reporting that exposure to ATV is not associated with increased risk for CV events.

PIs are a commonly used class of medications to treat HIV. The first medication within this class to be approved was saquinavir in 1995 [14]. Currently, DRV/r plus tenofovir/emtricitabine is listed as a recommended regimen and ATV/r plus tenofovir/emtricitabine is listed as an alternative regimen for treatment-naïve patients initiating ART [15]. However, PIs have been associated with dyslipidemia, though the pathways by which this occurs are not well understood [16]. In turn, dyslipidemia is associated with poor cardiovascular outcomes [17]. The same association with dyslipidemia may not exist for all medications within the PI class.

PI use has also been linked to increased risk for CV events in a number of analyses. A systematic review and meta-analysis by Bavinger et al. identified articles and abstracts published through 2011 [5]. They identified three analyses which evaluated cumulative use of PIs on the risk of MI [6, 18, 19]. The first, by Friis-Moller and colleagues, analyzed PIs as a medication class and found a significantly increased risk of MI with increased cumulative PI exposure [6]. Bavinger combined data from the two other analyses, by Worm et al. and Lang et al. and found that both cumulative indinavir use and cumulative lopinavir use was associated with increased risk for MI [18, 19]. Bavinger and colleagues also identified several studies which evaluated recent use of PIs and CV events [20–25]. MI was the most commonly evaluated CV outcome [21–25]. Three of five analyses reported increased risk of MI with recent PI exposure [22, 23, 25]. One conference abstract from 2010 by Triant and colleagues which analyzed specific PIs reported an increase risk of MI following exposure to nelfinavir or indinavir, specifically, but found no association with ATV use [26].

Two more recently published analyses by Monforte et al. and Stein et al. have also provided evidence against a link between ATV exposure and increased CV risk [7, 8]. Monforte and colleagues used D:A:D study data to evaluate the association between cumulative exposure to ATV and risk of MI or stroke [7]. MIs were rare (incidence rate per 100 person-years for patients unexposed to ATV = 0.28; for patients exposed to ATV for more than 3 years = 0.20), as were strokes (incidence rate per 100 person-years for patients unexposed to ATV = 0.17; for patients exposed to ATV for more than 3 years = 0.17) [7]. After controlling for demographics, clinical characteristics (including risk factors such as body mass index and smoking status), and exposure to other antiretroviral medications in Poisson regression, there was no evidence of an association between cumulative exposure of ATV and MI (relative rate/year = 0.95, 95 % CI 0.87-1.05) or stroke (relative rate/year = 0.95, 95 % CI 0.87-1.05) [7]. In ACTG 5260s, Stein and colleagues evaluated carotid intima-media thickness (IMT), which is a surrogate marker of CV risk, in treatment-naïve patients randomized to treatment with ATV/r plus tenofovir/emtricitabine, DRV/r plus tenofovir/emtricitabine, or raltegravir (an integrase inhibitor) plus tenofovir/emtricitabine [8]. Participants were followed for 144 weeks [8]. During follow-up, non-high-density lipoprotein cholesterol and triglycerides increased modestly in the ATV/r and DRV/r arms, and carotid IMT measurements increased in all arms during follow-up [8]. Carotid IMT progressed significantly more slowly in participants treated with ATV/r compared with patients treated with DRV/r [8]. The difference was even greater in patients who remained on their regimen throughout the follow-up period [8]. The comparison with raltegravir also favored ATV/r but was only statistically significant at the carotid bifurcation and in the on-treatment analyses [8]. Carotid IMT is a well-validated surrogate marker of atherosclerosis that has been shown to be an independent predictor of stroke and myocardial infarction [27–29].

Although the reason for the lack of association between ATV and increased CV events is unclear, one potential mechanism may be ATV-induced hyperbilirubinemia [30]. Bilirubin is known to have anti-atherosclerotic properties [31], and multiple studies have shown that individuals with Gilbert’s syndrome, a genetic deficiency in UGT1A1 resulting in a chronic low level hyperbilirubinemia, have a reduced risk for CV events [32, 33]. In ACTG 5260s, higher levels of on-treatment bilirubin were generally associated with slower progression of atherosclerosis [8].

This database analysis has several limitations and strengths. The limitations include those common to claims-based analyses. Claims are not collected for research purposes. Therefore, miscoding may occur that is non-differential by study cohorts, resulting in misclassification of ATV use or CV events. Regarding antiretroviral exposure, patients are assumed to take their medications as directed. The validity of this assumption is unknown. Regarding study outcomes, previously validated algorithms for MI and stroke [9, 10] were used to reduce the possibility of misclassification. Several patient characteristics related to CV risk, including information about viral load, CD4 count, family history, diet, exercise, smoking, and laboratory values, were not available in these datasets. If these factors were associated with both treatment and CV events, the results may be biased due to uncontrolled confounding. Additionally, patients may have been taking other ARV or non-ARV medications that could affect CV outcomes. Other than use of antihypertensive medications and antihyperlipidemic medications in baseline, this analysis did not control for the use of other medications. Provider information, including prescribing preferences were also not available in the claims data. As expected in a real-world analysis, there were small but significant differences between cohorts in some baseline characteristics such as gender, race, and prevalence of renal disease. This reflects differing provider preferences when prescribing ARV medications and was adjusted for using propensity-score weighted models. Given the potential link between abacavir exposure and increased CV risk [34], a Breslow-Day test for effect measure modification was conducted to determine if use of abacavir modified the relationship between ATV and CV events. The test was not significant. However, the test may have been underpowered due to the small number of events observed in this analysis, as effect modification has been noted in other claim-based analyses [35]. The small number of events may have also resulted in the study being underpowered to detect differences between the study cohorts, despite the large sample sizes. Lastly, patients may not have been followed long enough to develop these severe manifestations of CV disease, which may have resulted in the low prevalence of CV events. A claims-based analysis with longer follow-up would be helpful to confirm the lack of association between ATV use and CV events which was found here. The strengths of this analysis include two large samples of patients insured through a variety of plans. These patients represent a geographically and socioeconomically diverse group of HIV+ individuals. Additionally, this analysis is US-based, whereas the majority of previous data on CV events comes from D:A:D which is primarily based in Europe [36]. Lastly, this analysis includes more recent data which allows for comparisons with more contemporary ARVs.

## Conclusions

The results of this analysis are consistent with prior findings that there is no association between ATV exposure and increased risk for CV events. While other research indicates that the use of certain PIs increases risk for these events, it does not appear to be a universal class effect. This is an important consideration when selecting the appropriate HIV regimen for patients at increased risk of CV disease.

## Additional file

Additional file 1: ATV CV Events BMC Infectious Diseases Supplemental Files 09072016. **Table S1**. Codes to identify percutaneous coronary intervention and coronary artery bypass graft. **Table S2**. Demographic and baseline clinical characteristics of commercially-insured antiretroviral-naïve HIV+ patients initiating atazanavir-containing vs. atazanavir-free regimens. **Table S3**. Demographic and baseline clinical characteristics of Medicaid-insured antiretroviral-naïve HIV+ patients initiating atazanavir-containing vs. atazanavir-free regimens. **Table S4A**. Unadjusted incidence rates for CV events among commercially-insured HIV+ patients initiating atazanavir-containing vs. atazanavir-free regimens. **Table S4B**. Unadjusted incidence rates for CV events among Medicaid-insured HIV+ patients initiating atazanavir-containing vs. atazanavir-free regimens. **Table S5**. Propensity-score-weighted hazard ratios for CV events among antiretroviral-naïve HIV+ patients initiating atazanavir-containing vs. atazanavir-free regimens. (DOCX 36 kb)

## Abbreviations

ART: Antiretroviral therapy
ARV: Antiretroviral
ATV: Atazanavir
ATV/r: Ritonavir-boosted atazanavir
CABG: Coronary artery bypass graft
CI: Confidence interval
CV: Cardiovascular
D:A:D: Data Collection on Adverse Events of Anti-HIV Drugs
DRV/r: Ritonavir-boosted darunavir
HIPAA: Health Insurance Portability and Accountability Act
HIV: Human immunodeficiency virus
ICD-9-CM: *International Classification of Diseases, Ninth Edition, Clinical Modification*
IMT: Intima-media thickness
IR: Incidence rate
ITT: Intent-to-treat
MI: Myocardial infarction
NNRTI: Non-nucleoside reverse transcriptase inhibitor
PCI: Percutaneous coronary intervention
PI: Protease inhibitors
