# Erratum to: Risk of cardiovascular events among patients with HIV treated with atazanavircontaining regimens: a retrospective cohort study

**DOI:** 10.1186/s12879-016-1903-6

**Published:** 2016-10-07

**Authors:** Lisa Rosenblatt, Amanda M. Farr, Ella T. Nkhoma, James K. Nelson, Corey Ritchings, Stephen S. Johnston

**Affiliations:** 1 Bristol-Myers Squibb, 777 Scudders Mill Road, Plainsboro, NJ 08536 USA; 2 Truven Health Analytics, 150 Cambridgepark Drive, Cambridge, MA 02140 USA; 3 Bristol-Myers Squibb, 5 Research Parkway, Wallingford, CT 06492 USA; 4 Truven Health Analytics, 100 Phoenix Drive, Ann Arbor, MI 48108 USA; 5 Bristol-Myers Squibb, PO Box 4500, Princeton, NJ 08540 USA; 6 Truven Health Analytics, 7700 Old Georgetown Road, Bethesda, MD 20814 USA

## Erratum

*n.b. The errors and associated corrections described in this document concerning the original manuscript were accountable to the production department handling this manuscript, and thus are no fault of the authors of this paper. Additionally, the online manuscript has now been updated with these corrections accordingly.*

In the original publication of this article [1], Fig. 3 had [VALUE] (0.72, 2.23) where it should have said 1.27 (0.72, 2.23). This has now been updated in the original article.

**Fig. 3:**
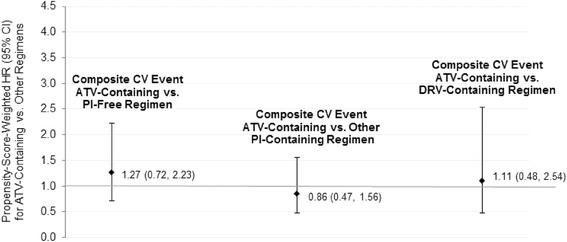
Propensity-score-weighted hazard ratios for composite CV event during as-treated follow-up period: Secondary comparisons. *CI* confidence interval, *CV* cardiovascular, *DRV* darunavir, *HR* hazard ratio, *PI* protease inhibitor

## References

[1] Rosenblatt L (2016). Risk of cardiovascular events among patients with HIV treated with atazanavircontaining regimens: a retrospective cohort study. BMC Infect Dis.

