# Supplementary material for: Risk of cardiovascular events among patients with HIV treated with atazanavir-containing regimens: a retrospective cohort study
Source: BMC Infect Dis. 2016 Sep 19;16:492. doi: 10.1186/s12879-016-1827-1 (PMC5028993; doi:10.1186/s12879-016-1827-1)
Supplement: Additional file 1: — ATV CV Events BMC Infectious Diseases Supplemental Files 09072016. Table S1. Codes to identify percutaneous coronary intervention and coronary artery bypass graft. Table S2. Demographic and baseline clinical characteristics of commercially-insured antiretroviral-naïve HIV+ patients initiating atazanavir-containing vs. atazanavir-free regimens. Table S3. Demographic and baseline clinical characteristics of Medicaid-insured antiretroviral-naïve HIV+ patients initiating atazanavir-containing vs. atazanavir-free regimens. Table S4A. Unadjusted incidence rates for CV events among commercially-insured HIV+ patients initiating atazanavir-containing vs. atazanavir-free regimens. Table S4B. Unadjusted incidence rates for CV events among Medicaid-insured HIV+ patients initiating atazanavir-containing vs. atazanavir-free regimens. Table S5. Propensity-score-weighted hazard ratios for CV events among antiretroviral-naïve HIV+ patients initiating atazanavir-containing vs. atazanavir-free regimens. (DOCX 36 kb) [file 12879_2016_1827_MOESM1_ESM.docx]

Table S1. Codes to identify percutaneous coronary intervention and coronary artery bypass graft

| **Outcome** | **Code List** |
| --- | --- |
| Percutaneous coronary intervention | ICD-9-CM procedure codes: 00.66, 36.06, 36.07, 17.55  CPT/HCPCS codes: G0290, G0291, 92980, 92981, 92982, 92984, 92995, 92996 |
| Coronary artery bypass graft | ICD-9-CM procedure codes: 36.10, 36.11, 36.12, 36.13, 36.14, 36.15, 36.16, 36.17, 36.19  CPT/HCPCS codes: 33510, 33511, 33512, 33513, 33514, 33516, 33517, 33518, 33519, 33521, 33522, 33523, 33530, 33533, 33534, 33535, 33536, S2205, S2206, S2207, S2208, S2209 |

ICD-9-CM, International Classification of Diseases, Ninth Edition, Clinical Modification; CPT, Current Procedural Terminology^®^; HCPCS, Healthcare Common Procedure Coding System.

Table S2. Demographic and baseline clinical characteristics of commercially-insured antiretroviral-naïve HIV+ patients initiating atazanavir-containing vs. atazanavir-free regimens

|  | **Commercial Database** | | | | | | | | | | |
| --- | --- | --- | --- | --- | --- | --- | --- | --- | --- | --- | --- |
|  | **ATV-Containing Regimen**  **N = 2,437** | | **PI-Free**  **Regimen**  **N = 16,131** | | **p-value** | **Other PI-Containing Regimen**  **N=3,463** | | **p-value** | **DRV-Containing Regimen**  **N=1,551** | | **p-value** |
|  | **N** | **%** | **N** | **%** |  | **N** | **%** |  | **N** | **%** |  |
| Age in Years  (Mean, SD) | 41.0 | 10.2 | 40.1 | 10.6 | 0.0001 | 41.5 | 10.0 | 0.0476 | 41.5 | 10.4 | 0.1327 |
| Male | 1,863 | 76.4% | 13,840 | 85.8% | <.0001 | 2,726 | 74.8% | 0.1507 | 1,264 | 81.5% | 0.0002 |
| Region |  |  |  |  |  |  |  |  |  |  |  |
| Northeast | 439 | 18.0% | 2,612 | 16.2% | 0.0001 | 534 | 14.7% | 0.0012 | 245 | 15.8% | 0.4244 |
| North Central | 290 | 11.9% | 2,436 | 15.1% |  | 460 | 12.6% |  | 182 | 11.7% |  |
| South | 1,229 | 50.4% | 8,183 | 50.7% |  | 1,991 | 54.7% |  | 798 | 51.5% |  |
| West | 440 | 18.1% | 2,690 | 16.7% |  | 610 | 16.7% |  | 301 | 19.4% |  |
| Unknown | 39 | 1.6% | 210 | 1.3% |  | 48 | 1.3% |  | 25 | 1.6% |  |
| Capitation | 548 | 22.5% | 3,025 | 18.8% | <.0001 | 729 | 20.0% | 0.0202 | 314 | 20.2% | 0.0936 |
| CHADS_2_ Score |  |  |  |  |  |  |  |  |  |  |  |
| 0 | 2,040 | 83.7% | 13,372 | 82.9% | 0.3805 | 3,010 | 82.6% | 0.2097 | 1,257 | 81.0% | 0.0179 |
| 1 | 321 | 13.2% | 2,243 | 13.9% |  | 491 | 13.5% |  | 225 | 14.5% |  |
| 2 | 67 | 2.7% | 420 | 2.6% |  | 115 | 3.2% |  | 53 | 3.4% |  |
| 3–6 | 9 | 0.4% | 96 | 0.6% |  | 27 | 0.7% |  | 16 | 1.0% |  |
| Diabetes Mellitus | 115 | 4.7% | 780 | 4.8% | 0.8024 | 181 | 5.0% | 0.6577 | 80 | 5.2% | 0.5308 |
| Hypertension | 370 | 15.2% | 2,666 | 16.5% | 0.0944 | 590 | 16.2% | 0.2885 | 283 | 18.2% | 0.0108 |
| Dyslipidemia | 306 | 12.6% | 2,011 | 12.5% | 0.9006 | 462 | 12.7% | 0.8853 | 196 | 12.6% | 0.9404 |
| Renal Disease | 107 | 4.4% | 455 | 2.8% | <.0001 | 185 | 5.1% | 0.2191 | 98 | 6.3% | 0.0072 |
| Tobacco Use Disorder | 110 | 4.5% | 818 | 5.1% | 0.2393 | 168 | 4.6% | 0.8580 | 82 | 5.3% | 0.2662 |
| COPD | 37 | 1.5% | 219 | 1.4% | 0.5262 | 60 | 1.6% | 0.6946 | 22 | 1.4% | 0.7991 |
| Anemia | 186 | 7.6% | 1,302 | 8.1% | 0.4568 | 408 | 11.2% | <.0001 | 221 | 14.2% | <.0001 |
| Hepatitis C | 67 | 2.7% | 333 | 2.1% | 0.0300 | 86 | 2.4% | 0.3431 | 38 | 2.5% | 0.5650 |
| Alcohol Abuse Disorder | 14 | 0.6% | 125 | 0.8% | 0.2847 | 22 | 0.6% | 0.8835 | 12 | 0.8% | 0.4460 |
| Drug Abuse Disorder | 137 | 5.6% | 1,026 | 6.4% | 0.1607 | 224 | 6.1% | 0.3940 | 112 | 7.2% | 0.0418 |
| Autoimmune/Infla-mmatory Disorders | 86 | 3.5% | 602 | 0.0% | 0.6209 | 129 | 0.0% | 0.9800 | 56 | 0.0% | 0.8921 |
| Circulatory Disease | 504 | 20.7% | 3,614 | 22.4% | 0.0564 | 856 | 23.5% | 0.0098 | 408 | 26.3% | <.0001 |
| Oral Contraceptives | 25 | 1.0% | 104 | 0.6% | 0.0347 | 42 | 1.2% | 0.6419 | 11 | 0.7% | 0.3027 |

ATV, atazanavir; COPD, chronic obstructive pulmonary disorder; DRV, darunavir; PI, protease inhibitor.

Table S3. Demographic and baseline clinical characteristics of Medicaid-insured antiretroviral-naïve HIV+ patients initiating atazanavir-containing vs. atazanavir-free regimens

|  | **Medicaid Database** | | | | | | | | | | |
| --- | --- | --- | --- | --- | --- | --- | --- | --- | --- | --- | --- |
|  | **ATV-Containing Regimen**  **N = 1,505** | | **PI-Free**  **Regimen**  **N = 3,931** | | **p-value** | **Other PI-Containing Regimen**  **N=1,700** | | **p-value** | **DRV-Containing Regimen**  **N=527** | | **p-value** |
|  | **N** | **%** | **N** | **%** |  | **N** | **%** |  | **N** | **%** |  |
| Age in Years  (Mean, SD) | 41.2 | 10.9 | 41.9 | 11.0 | 0.0325 | 39.9 | 11.5 | 0.0021 | 42.4 | 10.5 | 0.0297 |
| Male | 746 | 49.6% | 2,071 | 52.7% | 0.0792 | 812 | 47.8% | 0.1076 | 299 | 56.7% | 0.0002 |
| Race |  |  |  |  |  |  |  |  |  |  |  |
| White | 206 | 13.7% | 680 | 17.3% | 0.0169 | 284 | 16.7% | 0.1005 | 91 | 17.3% | 0.0532 |
| Black | 1,088 | 72.3% | 2,714 | 69.0% |  | 1,180 | 69.4% |  | 346 | 65.7% |  |
| Hispanic | 16 | 1.1% | 50 | 1.3% |  | 26 | 1.5% |  | 8 | 1.5% |  |
| Other | 13 | 0.9% | 44 | 1.1% |  | 18 | 1.1% |  | 8 | 1.5% |  |
| Unknown/  Missing | 182 | 12.1% | 443 | 11.3% |  | 192 | 11.3% |  | 74 | 14.0% |  |
| Capitation | 609 | 40.5% | 1,492 | 38.0% | 0.0890 | 764 | 44.9% | 0.0106 | 162 | 30.7% | <.0001 |
| CHADS_2_ Score |  |  |  |  |  |  |  |  |  |  |  |
| 0 | 1,019 | 67.7% | 2,555 | 65.0% | 0.1750 | 1,190 | 70.0% | 0.4479 | 324 | 61.5% | 0.0237 |
| 1 | 330 | 21.9% | 959 | 24.4% |  | 335 | 19.7% |  | 140 | 26.6% |  |
| 2 | 113 | 7.5% | 318 | 8.1% |  | 130 | 7.6% |  | 52 | 9.9% |  |
| 3–6 | 43 | 2.9% | 99 | 2.5% |  | 45 | 2.7% |  | 11 | 2.1% |  |
| Diabetes Mellitus | 137 | 9.1% | 400 | 10.2% | 0.2357 | 163 | 9.6% | 0.6379 | 66 | 12.5% | 0.0242 |
| Hypertension | 450 | 29.9% | 1,280 | 32.6% | 0.0594 | 453 | 26.6% | 0.0410 | 175 | 33.2% | 0.1569 |
| Dyslipidemia | 158 | 10.5% | 429 | 10.9% | 0.6592 | 147 | 8.6% | 0.0747 | 57 | 10.8% | 0.8384 |
| Renal Disease | 146 | 9.7% | 343 | 8.7% | 0.2607 | 181 | 10.6% | 0.3772 | 74 | 14.0% | 0.0058 |
| Tobacco Use Disorder | 347 | 23.1% | 887 | 22.6% | 0.6983 | 370 | 21.8% | 0.3811 | 155 | 29.4% | 0.0036 |
| COPD | 88 | 5.8% | 291 | 7.4% | 0.0439 | 101 | 5.9% | 0.9102 | 38 | 7.2% | 0.2640 |
| Anemia | 283 | 18.8% | 737 | 18.7% | 0.9625 | 351 | 20.6% | 0.1911 | 138 | 26.2% | 0.0003 |
| Hepatitis C | 173 | 11.5% | 409 | 10.4% | 0.2446 | 186 | 10.9% | 0.6198 | 54 | 10.2% | 0.4337 |
| Alcohol Abuse Disorder | 90 | 6.0% | 160 | 4.1% | 0.0026 | 86 | 5.1% | 0.2532 | 34 | 6.5% | 0.6971 |
| Drug Abuse Disorder | 488 | 32.4% | 1,159 | 29.5% | 0.0347 | 507 | 29.8% | 0.1121 | 195 | 37.0% | 0.0556 |
| Autoimmune/Infla-mmatory Disorders | 80 | 5.3% | 213 | 0.1% | 0.8806 | 71 | 0.0% | 0.1288 | 28 | 0.1% | 0.9982 |
| Circulatory Disease | 570 | 37.9% | 1,559 | 39.6% | 0.2276 | 619 | 36.4% | 0.3925 | 233 | 44.2% | 0.0104 |
| Oral Contraceptives | 13 | 0.9% | 30 | 0.7% | 0.7079 | 12 | 0.7% | 0.6121 | 2 | 0.4% | 0.3797 |

ATV, atazanavir; COPD, chronic obstructive pulmonary disorder; DRV, darunavir; PI, protease inhibitor.

Table S4A. Unadjusted incidence rates for CV events among commercially-insured HIV+ patients initiating atazanavir-containing vs. atazanavir-free regimens

|  | **ATV-Containing Regimen** | **ATV-Free Regimen** | **PI-Free**  **Regimen** | **Other PI-Containing Regimen** | **DRV-Containing Regimen** |
| --- | --- | --- | --- | --- | --- |
| ***As-Treated Follow-Up*** | | | | | |
| ***MI*** |  | |  |  |  |
| Number of Events | 3 | 26 | 17 | 9 | 2 |
| Crude IR per 1,000 PYs (95% CI) | 1.29  (0.27-3.76) | 1.24  (0.81-1.82) | 0.95  (0.55-1.52) | 3.13  (1.43-5.94) | 1.61  (0.20-5.81) |
| ***Stroke*** |  | |  |  |  |
| Number of Events | 2 | 21 | 16 | 5 | 2 |
| Crude IR per 1,000 PYs (95% CI) | 0.86  (0.10-3.10) | 1.00  (.62-1.54) | 0.89  (0.51-1.45) | 1.73  (0.56-4.04) | 1.61  (0.19-5.82) |
| ***PCI*** |  | |  |  |  |
| Number of Events | 2 | 29 | 19 | 10 | 2 |
| Crude IR per 1,000 PYs (95% CI) | 0.86  (0.10-3.10) | 1.39  (0.93-1.99) | 1.06  (0.64-1.66) | 3.48  (1.67-6.41) | 1.61  (0.20-5.81) |
| ***CABG*** |  | |  |  |  |
| Number of Events | 1 | 14 | 13 | 1 | 1 |
| Crude IR per 1,000 PYs (95% CI) | 0.43  (0.01-2.39) | 0.67  (0.37-1.12) | 0.73  (0.39-1.24) | 0.35  (0.009-1.93) | 0.80  (0.02-4.48) |
| ***Composite CV Endpoint*** |  | |  |  |  |
| Number of Events | 7 | 68 | 49 | 19 | 5 |
| Crude IR per 1,000 PYs (95% CI) | 3.01  (1.21-6.21) | 3.26  (2.53-4.13) | 2.74  (2.03-3.63) | 6.63  (3.99-10.36) | 4.03  (1.31-9.40) |
| ***ITT Follow-Up*** | | | | | |
| ***MI*** |  | |  |  |  |
| Number of Events | 8 | 51 | 34 | 17 | 3 |
| Crude IR per 1,000 PYs (95% CI) | 1.67  (0.72-3.29) | 1.47  (1.10-1.94) | 1.21  (0.84-1.69) | 2.59  (1.51-4.14) | 1.32  (0.27-3.85) |
| ***Stroke*** |  | |  |  |  |
| Number of Events | 10 | 45 | 36 | 9 | 3 |
| Crude IR per 1,000 PYs (95% CI) | 2.08  (1.00-3.83) | 1.30  (0.95-1.74) | 1.28  (0.90-1.78) | 1.36  (0.62-2.59) | 1.32  (0.27-3.84) |
| ***PCI*** |  | |  |  |  |
| Number of Events | 4 | 62 | 37 | 25 | 3 |
| Crude IR per 1,000 PYs (95% CI) | 0.83  (0.23-2.13) | 1.79  (1.37-2.30) | 1.32  (0.93-1.82) | 3.82  (2.47-5.63) | 1.32  (0.27-3.85) |
| ***CABG*** |  | |  |  |  |
| Number of Events | 2 | 24 | 19 | 5 | 2 |
| Crude IR per 1,000 PYs (95% CI) | 0.42  (0.05-1.50) | 0.69  (0.44-1.03) | 0.68  (0.41-1.06) | 0.76  (0.25-1.77) | 0.88  (0.11-3.17) |
| ***Composite CV Endpoint*** |  | |  |  |  |
| Number of Events | 20 | 136 | 95 | 41 | 8 |
| Crude IR per 1,000 PYs (95% CI) | 4.19  (2.56-6.47) | 3.94  (3.28-4.60) | 3.40  (2.75-4.15) | 6.28  (4.51-8.52) | 3.52  (1.52-6.94) |

ATV, atazanavir; CABG, coronary artery bypass graft; CI, confidence interval; CV, cardiovascular; DRV, darunavir; IR, incidence rate; ITT, intent-to-treat; MI, myocardial infarction; PCI, percutaneous coronary intervention; PI, protease inhibitors; PY, person-years.

Table S4B. Unadjusted incidence rates for CV events among Medicaid-insured HIV+ patients initiating atazanavir-containing vs. atazanavir-free regimens

|  | **ATV-Containing Regimen** | **ATV-Free Regimen** | **PI-Free**  **Regimen** | **ATV-Free PI-Containing Regimen** | **DRV-Containing Regimen** |
| --- | --- | --- | --- | --- | --- |
| ***As-Treated Follow-Up*** | | | | | |
| ***MI*** |  | |  |  |  |
| Number of Events | 1 | 15 | 10 | 5 | 1 |
| Crude IR per 1,000 PYs (95% CI) | 0.91  (0.02-5.04) | 3.79  (2.12-6.26) | 3.37  (1.62-6.20) | 5.18  (1.68-12.09) | 3.62  (0.09-20.17) |
| ***Stroke*** |  | |  |  |  |
| Number of Events | 8 | 19 | 15 | 4 | 2 |
| Crude IR per 1,000 PYs (95% CI) | 7.29  (3.15-14.37) | 4.81  (2.90-7.51) | 5.07  (2.84-8.35) | 4.13  (1.13-10.57) | 7.25  (0.88-26.18) |
| ***PCI*** |  | |  |  |  |
| Number of Events | 2 | 8 | 6 | 2 | 0 |
| Crude IR per 1,000 PYs (95% CI) | 1.81  (0.22-6.54) | 2.02  (0.87-3.98) | 2.02  (0.74-4.40) | 2.07  (0.25-7.48) | 0.0  (0.0-13.35) |
| ***CABG*** |  | |  |  |  |
| Number of Events | 1 | 3 | 1 | 2 | 0 |
| Crude IR per 1,000 PYs (95% CI) | 0.91  (0.02-5.04) | 0.76  (0.16-2.21) | 0.34  (0.008-1.87) | 2.06  (0.25-7.45) | 0.0  (0.0-13.35) |
| ***Composite CV Endpoint*** |  | |  |  |  |
| Number of Events | 12 | 39 | 29 | 10 | 3 |
| Crude IR per 1,000 PYs (95% CI) | 10.94  (5.66-19.12) | 9.92  (7.06-13.56) | 9.85  (6.59-14.14) | 10.37  (4.97-19.07) | 10.87  (2.24-31.77) |
| ***ITT Follow-Up*** | | | | | |
| ***MI*** |  | |  |  |  |
| Number of Events | 10 | 30 | 17 | 13 | 3 |
| Crude IR per 1,000 PYs (95% CI) | 3.53  (1.69-6.48) | 3.0  (2.02-4.28) | 2.50  (1.46-4.00) | 4.05  (2.16-6.92) | 3.70  (0.76-10.80) |
| ***Stroke*** |  | |  |  |  |
| Number of Events | 17 | 55 | 39 | 16 | 3 |
| Crude IR per 1,000 PYs (95% CI) | 6.0  (3.50-9.61) | 5.51  (4.15-7.17) | 5.76  (4.10-7.88) | 4.98  (2.85-8.09) | 3.70  (0.76-10.80) |
| ***PCI*** |  | |  |  |  |
| Number of Events | 5 | 15 | 11 | 4 | 1 |
| Crude IR per 1,000 PYs (95% CI) | 1.76  (0.57-4.10) | 1.50  (0.84-2.47) | 1.62  (0.81-2.90) | 1.24  (0.34-3.18) | 1.23  (0.03-6.85) |
| ***CABG*** |  | |  |  |  |
| Number of Events | 3 | 7 | 5 | 2 | 0 |
| Crude IR per 1,000 PYs (95% CI) | 1.05  (0.22-3.07) | 0.70  (0.28-1.44) | 0.74  (0.24-1.71) | 0.62  (0.08-2.24) | 0.0  (0.0-4.53) |
| ***Composite CV Endpoint*** |  | |  |  |  |
| Number of Events | 32 | 94 | 63 | 31 | 7 |
| Crude IR per 1,000 PYs (95% CI) | 11.41  (7.80-16.11) | 9.47  (7.66-11.59) | 9.36  (7.19-11.97) | 9.72  (0.60-13.79) | 8.66  (3.48-17.85) |

ATV, atazanavir; CABG, coronary artery bypass graft; CI, confidence interval; CV, cardiovascular; DRV, darunavir; IR, incidence rate; ITT, intent-to-treat; MI, myocardial infarction; PCI, percutaneous coronary intervention; PI, protease inhibitors; PY, person-years.

Table S5. Propensity-score-weighted hazard ratios for CV events among antiretroviral-naïve HIV+ patients initiating atazanavir-containing vs. atazanavir-free regimens

|  | **Hazard Ratio (95% CI) for CV Event** | | | |
| --- | --- | --- | --- | --- |
| **CV Event** | **ATV-Containing vs. ATV-Free Regimen** | **ATV-Containing vs. PI-Free Regimen** | **ATV-Containing vs. Other PI-Containing Regimen** | **ATV-Containing vs. DRV-Containing Regimen** |
| *As-Treated Follow-Up* | | | | |
| MI | 0.976 (0.330, 2.887) | 1.154 (0.381, 3.496) | 0.512 (0.158, 1.660) | 0.880 (0.192, 4.040) |
| Stroke | 1.321 (0.607, 2.874) | 1.415 (0.632, 3.164) | 1.247 (0.503, 3.096) | 1.166 (0.348, 3.905) |
| PCI | 0.753 (0.231, 2.454) | 0.956 (0.278, 3.284) | 0.415 (0.138, 1.247) | 0.718 (0.144, 3.573) |
| CABG | 0.932 (0.215, 4.049) | 0.862 (0.177, 4.192) | 0.984 (0.161, 6.012) | 0.841 (0.093, 7.615) |
| Composite CV Event | 1.155 (0.670, 1.991) | 1.268 (0.721, 2.229) | 0.856 (0.470, 1.562) | 1.105 (0.480, 2.544) |
| *ITT Follow-Up* | | | | |
| MI | 1.311 (0.738, 2.327) | 1.642 (0.892, 3.025) | 0.795 (0.429, 1.473) | 1.153 (0.424, 3.137) |
| Stroke | 1.284 (0.800, 2.062) | 1.295 (0.794, 2.111) | 1.353 (0.759, 2.412) | 2.552 (1.005, 6.481) |
| PCI | 0.700 (0.314, 1.557) | 0.902 (0.384, 2.117) | 0.421 (0.196, 0.905) | 0.731 (0.220, 2.427) |
| CABG | 0.718 (0.253, 2.035) | 0.671 (0.225, 1.999) | 1.023 (0.303, 3.457) | 0.896 (0.178, 4.505) |
| Composite CV Event | 1.142 (0.815, 1.602) | 1.272 (0.892, 1.814) | 0.930 (0.638, 1.357) | 1.436 (0.769, 2.682) |

ATV, atazanavir; CABG, coronary artery bypass graft; CI, confidence interval; CV, cardiovascular; DRV, darunavir; ITT, intent-to-treat; MI, myocardial infarction; PCI, percutaneous coronary intervention; PI, protease inhibitors.
